# Effectiveness of a COVID-19 Additional Primary or Booster Vaccine Dose in Preventing SARS-CoV-2 Infection Among Nursing Home Residents During Widespread Circulation of the Omicron Variant — United States, February 14–March 27, 2022

**DOI:** 10.15585/mmwr.mm7118a4

**Published:** 2022-05-06

**Authors:** Namrata Prasad, Gordana Derado, Srinivas Acharya Nanduri, Hannah E. Reses, Heather Dubendris, Emily Wong, Minn Minn Soe, Qunna Li, Philip Dollard, Suparna Bagchi, Jonathan Edwards, Nong Shang, Dan Budnitz, Jeneita Bell, Jennifer R. Verani, Andrea Benin, Ruth Link-Gelles, John Jernigan, Tamara Pilishvili

**Affiliations:** ^1^CDC COVID-19 Emergency Response Team; ^2^Epidemic Intelligence Service, CDC.

Nursing home residents have experienced disproportionally high levels of COVID-19–associated morbidity and mortality and were prioritized for early COVID-19 vaccination (*1*). Following reported declines in vaccine-induced immunity after primary series vaccination, defined as receipt of 2 primary doses of an mRNA vaccine (BNT162b2 [Pfizer-BioNTech] or mRNA-1273 [Moderna]) or 1 primary dose of Ad26.COV2 (Johnson & Johnson [Janssen]) vaccine (*2*), CDC recommended that all persons aged ≥12 years receive a COVID-19 booster vaccine dose.* Moderately to severely immunocompromised persons, a group that includes many nursing home residents, are also recommended to receive an additional primary COVID-19 vaccine dose.^†^ Data on vaccine effectiveness (VE) of an additional primary or booster dose against infection with SARS-CoV-2 (the virus that causes COVID-19) among nursing home residents are limited, especially against the highly transmissible B.1.1.529 and BA.2 (Omicron) variants. Weekly COVID-19 surveillance and vaccination coverage data among nursing home residents, reported by skilled nursing facilities (SNFs) to CDC’s National Healthcare Safety Network (NHSN)^§^ during February 14–March 27, 2022, when the Omicron variant accounted for >99% of sequenced isolates, were analyzed to estimate relative VE against infection for any COVID-19 additional primary or booster dose compared with primary series vaccination. After adjusting for calendar week and variability across SNFs, relative VE of a COVID-19 additional primary or booster dose was 46.9% (95% CI = 44.8%–48.9%). These findings indicate that among nursing home residents, COVID-19 additional primary or booster doses provide greater protection against Omicron variant infection than does primary series vaccination alone. All immunocompromised nursing home residents should receive an additional primary dose, and all nursing home residents should receive a booster dose, when eligible, to protect against COVID-19. Efforts to keep nursing home residents up to date with vaccination should be implemented in conjunction with other COVID-19 prevention strategies, including testing and vaccination of nursing home staff members and visitors.

Each week, nursing homes certified by Centers for Medicaid & Medicare Services (CMS) report incident confirmed SARS-CoV-2 infections among residents and staff members, by vaccination status, to NHSN. This study was limited to case data reported by CMS-certified SNFs, which account for >90% of nursing homes reporting COVID-19 data to NHSN, during February 14–March 27, 2022, when the Omicron variant accounted for >99% of sequenced isolates nationwide.^¶^ COVID-19 case ascertainment at CMS-certified SNFs during the study period was high, because of guidelines recommending weekly testing of all residents and staff members if a single SARS-CoV-2 infection was identified in a facility.** At SNFs with contact tracing capacity, only close contacts of an infected resident or staff member were tested. Vaccination status of infected persons was categorized as 1) vaccinated with a primary series only (receipt of 2 doses of an mRNA vaccine or 1 dose of the Janssen COVID-19 vaccine ≥14 days before a SARS-CoV-2–positive test result, or receipt of an additional primary or booster dose <14 days before a SARS-CoV-2–positive test result), 2) vaccinated with an additional or booster dose (receipt of any authorized COVID-19 additional primary or booster dose ≥14 days before a SARS-CoV-2–positive test result),^††^ 3) unvaccinated (no COVID-19 vaccine dose or a single dose <14 days before a SARS-CoV-2–positive test result), and 4) other (receipt of a single mRNA vaccine dose ≥14 days before a SARS-CoV-2–positive test result or unspecified vaccination).

SNFs also report the weekly census of residents by vaccination status. Resident-weeks were calculated as the aggregate of weekly resident counts, by vaccination status, at each SNF. In this study, weekly case counts by vaccination status in each SNF were paired with weekly resident counts by vaccination status from 2 weeks earlier. Data from SNFs that reported no additional or booster dose coverage throughout the study period were excluded.^§§^ In addition, weekly case count reports were excluded if a facility did not report corresponding resident counts for the preceding 2 weeks. Crude infection rates by vaccination status were calculated with 95% CIs based on the binominal distribution.

Infection rates among residents who received an additional or booster dose were compared with those who received primary series vaccination only to estimate relative additional or booster dose VE. The effectiveness of primary series vaccination or an additional or booster dose compared with no vaccination (i.e., absolute VEs) were not reported because of differences in visitation, quarantine, and masking policies between unvaccinated residents and vaccinated residents based on updated CMS guidelines,^¶¶^ as well as the inability to adjust for confounding because of these factors. Product-specific VE also could not be estimated because SNFs only reported relevant vaccine product information in weekly resident count reports and not within weekly case count reports.

A zero-inflated Poisson mixed effects model, which adjusted for calendar week using quadratic splines and included SNF as a random effect to account for variability across facilities, was used to estimate the ratio of infection rates between residents who received an additional or booster dose and those who received primary series vaccination only. Relative VE was estimated as 1 minus the rate ratio multiplied by 100. The following characteristics were evaluated as potential confounders of relative VE: 1) weekly cumulative staff member and resident SARS-CoV-2 infection rates at each SNF during the study period (since May 8, 2020), 2) weekly SNF-level staff member COVID-19 vaccination coverage, 3) each SNF’s county-level incidence of SARS-CoV-2 infection, and 4) each SNF county’s CDC Social Vulnerability Index score.*** A 10% change-in-estimate criterion for the regression coefficient was used to evaluate covariates; none met this criterion and thus none were included in the final model. Data analysis was conducted using SAS software (version 9.4; SAS Institute) and R software (version 4.1.2; R Foundation). This activity was reviewed by CDC and was conducted consistent with federal laws and institutional policies.^†††^

Overall, 15,090 SNFs provided 89,671 weekly case count reports during February 14–March 27, 2022, and 15,102 SNFs provided 89,969 weekly resident count reports during January 31–March 13, 2022. After applying exclusion criteria and pairing SNF-level weekly case with corresponding resident data, the analysis included 85,494 reports from 14,758 SNFs. The median weekly number of residents reported was 1,126,198 (IQR = 1,124,328–1,126,709); approximately 22% of whom had received primary series vaccination only, and 65% of whom had received an additional or booster dose. Among residents who had received primary series vaccination or an additional or booster dose, >90% had received mRNA COVID-19 vaccines.

Crude weekly confirmed SARS-CoV-2 infection rates declined across all vaccination groups during the study period (Figure); however, rates of infection among residents with an additional or booster dose were consistently lower than those among residents with primary series vaccination only or among unvaccinated residents. Overall, 7,510 cases were confirmed among 1,509,674 resident-weeks with primary series vaccination only and 11,334 cases were confirmed among 4,416,401 resident-weeks with an additional or booster dose (Table). The adjusted relative VE against infection for an additional or booster dose versus primary series vaccination only was 46.9%.

**FIGURE:**
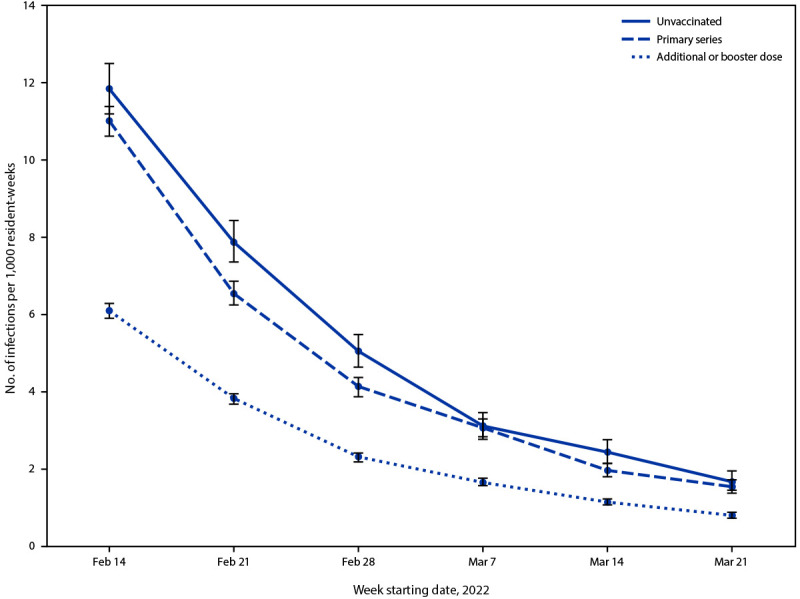
Crude weekly rates of reported confirmed SARS-CoV-2 infection among skilled nursing facility residents,* by vaccination status^†^ and resident-week^§^ — National Healthcare Safety Network, United States, February 14–March 27, 2022 * Crude rates of SARS-CoV-2 infection were calculated as the number of cases, by vaccination status, among residents with corresponding vaccination status from 2 weeks earlier (January 31–March 13, 2022); 95% CIs indicated by error bars. ^†^ Residents who completed a primary vaccination series were those who received 2 primary doses of an mRNA vaccine (Pfizer-BioNTech or Moderna) or 1 primary dose of Janssen vaccine. Residents with additional or booster dose vaccination were those who received an additional primary vaccine dose ≥28 days after the initial primary series or a booster dose ≥5 months after completion of an mRNA primary series or ≥2 months after 1 primary Janssen vaccine dose. Residents with an additional or booster dose included those who received additional primary vaccine doses and a booster dose. Unvaccinated residents were those who received no COVID-19 vaccine doses. Cases among residents with primary series vaccination were defined as infections in residents who had received primary series vaccination ≥14 days before a SARS-CoV-2–positive test result or received an additional or booster dose <14 days before a SARS-CoV-2–positive test result. Cases among residents with additional or booster dose vaccination were defined as infections in residents who received an additional primary or booster dose ≥14 days before a SARS-CoV-2–positive test result. Cases among unvaccinated residents were defined as infections in residents who received no COVID-19 vaccine doses or a single dose <14 days before a SARS-CoV-2–positive test result. Data on infections among residents who received a single dose of mRNA vaccine ≥14 days before a SARS-CoV-2–positive test result or unspecified vaccination are not presented. ^§^ Resident-weeks were calculated as the aggregate of weekly resident counts, by vaccination status, at each skilled nursing facility.

**TABLE:** Relative effectiveness of additional COVID-19 primary or booster vaccine doses in preventing SARS-CoV-2 infection among residents of skilled nursing facilities compared with primary series vaccination only — National Healthcare Safety Network, United States, February 14–March 27, 2022

Vaccination status*	No. of resident-weeks^†^	No. of cases^§^	Crude infection rate^¶^ (95% CI)	Vaccine effectiveness % (95% CI)	
				Unadjusted**	Adjusted^††^
**Primary series **	1,509,674	7,510	5.0 (4.9–5.1)	Ref	Ref
**Additional or booster dose**	4,416,401	11,334	2.6 (2.5–2.7)	49.3 (47.3–51.3)	46.9 (44.8–48.9)

**Abbreviations**: Ref = referent group; SNF = skilled nursing facility.

* Residents who completed a primary vaccination series were those who received 2 primary doses of an mRNA vaccine Pfizer-BioNTech or Moderna or 1 primary dose of Janssen vaccine. Residents with additional or booster dose vaccination were those who received an additional primary vaccine dose ≥28 days after the initial primary series or a booster dose ≥5 months after completion of an mRNA primary series or ≥2 months after 1 primary Janssen vaccine dose. Residents with an additional or booster dose included those who received additional primary vaccine doses and a booster dose.

^†^ Resident-weeks were calculated as the aggregate of weekly resident counts, by vaccination status, at each SNF.

^§^ Cases among residents with primary series vaccination were defined as infections in residents who had received primary series vaccination ≥14 days before a SARS-CoV-2–positive test result or received an additional or booster dose <14 days before a positive SARS-CoV-2 test result. Cases among residents with additional or booster dose vaccination were defined as infections in residents who had received an additional primary or booster dose ≥14 days before a positive SARS-CoV-2 test result. For analysis, weekly case counts by vaccination status in each SNF were paired with weekly resident counts by vaccination status from 2 weeks earlier (January 31–March 13, 2022).

^¶^ Infections per 1,000 resident-weeks.

** Results from a zero-inflated Poisson mixed effects model with random effects for SNF. Vaccine effectiveness was estimated as 1 minus the rate ratio multiplied by 100, with the rate ratio comparing infection rates among residents vaccinated with an additional or booster dose to residents with primary series vaccination only.

**^††^** Results from the same model while also controlling for calendar week using quadratic splines.

## Discussion

Analysis of NHSN’s COVID-19 surveillance and vaccination coverage data from 14,758 SNFs, including approximately 1 million nursing home residents, found that additional or booster COVID-19 vaccine doses provide greater protection against infection with the Omicron variant compared with primary series vaccination alone. Efforts to keep nursing home residents up to date^§§§^ with vaccination should be implemented in conjunction with other COVID-19 prevention strategies, including testing and vaccination of nursing home staff members and visitors.

The findings from this study are typically consistent with previous research, including a study among two nursing home systems in the United States during SARS-CoV-2 B.1.617.2 (Delta) variant predominance, in which the relative effectiveness of a COVID-19 mRNA booster dose against infection, compared with primary series vaccination alone, was reported to range from 50.4% to 58.2%.^¶¶¶^ Relative VE estimates in this study are slightly lower and might reflect declines in VE because of potential immune evasion of the Omicron variant, consistent with findings from other studies that indicated lower VE against Omicron variant infection compared with Delta variant infection among adults aged ≥18 years (*3**,**4*). In addition, although VE by time since vaccination could not be evaluated in this study, NHSN vaccination coverage data indicate that >50% of nursing home residents had received an additional or booster dose by early December 2021****; thus, the potential waning of vaccine-induced immunity with time since an additional or booster dose receipt might also have contributed to the lower VE estimates observed in this study.

The findings in this report are subject to at least six limitations. First, NHSN does not receive resident-level demographic or clinical data. Therefore, the analysis could not account for time since vaccination, nor could it control for potential confounders, such as age, comorbidities, previous SARS-CoV-2 infection, or behaviors related to SARS-CoV-2 infection risk (e.g., mask use). Second, the analysis could not distinguish between immunocompromised residents who received an additional primary dose and residents who received a booster dose, nor could it seperate residents who received both an additional primary and booster dose. Third, differences in visitation, quarantine, and masking policies between unvaccinated residents and vaccinated residents precluded estimation of absolute VE of primary series vaccination or additional or booster doses. Fourth, relevant vaccine product data were not collected, and, therefore, product specific VE could not be estimated. Fifth, residents were only considered to be protected with an additional or booster dose 14 days after receipt of their last dose, and SARS-CoV-2 infections among residents with primary series vaccination included infections among residents who had received an additional or booster dose <14 days earlier; protective effects of these additional or booster doses might begin sooner than 14 days and, therefore, categorization of such residents and cases within the primary series vaccination group might have biased relative VE estimates in this study. Finally, this analysis was unable to distinguish between asymptomatic and symptomatic infections or assess VE of an additional or booster dose against more severe COVID-19–associated outcomes. Recent studies have reported effectiveness of a third COVID-19 mRNA dose, compared with no vaccination, to range between 80% and 90% against medically attended COVID-19–associated outcomes during Omicron variant predominance^††††^ (*5*).

Efforts to maximize vaccination coverage, including additional primary doses, if recommended, and a booster dose, when eligible, among nursing home residents are critical. Such efforts should be implemented in conjunction with other COVID-19 prevention strategies, including testing and vaccination of nursing home staff members and visitors. The Food and Drug Administration has recently authorized a second booster dose for all adults aged ≥50 years and for persons aged ≥12 years who are moderately or severely immunocompromised.^§§§§^ This authorization was based on data from Israel illustrating increased protection from a fourth mRNA vaccine dose against SARS-CoV-2 infection and severe COVID-19 (*6*). Ongoing monitoring of VE of additional or booster doses among nursing home residents is critical to assess the durability of protection provided by such strategies and the effectiveness against emerging SARS-CoV-2 variants.

**Summary**

**What is already known about this topic?**

Nursing home residents are at high risk for COVID-19–associated morbidity and mortality. Little is known about the vaccine effectiveness (VE) of additional or booster COVID-19 vaccine doses against SARS-CoV-2 infection in this population, particularly against the Omicron variant.

**What is added by this report?**

Analysis of COVID-19 surveillance and vaccination data from approximately 15,000 skilled nursing facilities found that, compared with primary series vaccination only, an additional or booster dose provided greater protection (relative VE = 46.9%) against SARS-CoV-2 infection during Omicron variant predominance.

**What are the implications for public health practice?**

All immunocompromised nursing home residents should receive an additional primary dose, and all nursing home residents should receive a booster dose, when eligible, to protect against COVID-19.

## Footnotes

* During September–October, 2021, CDC recommended use of a single COVID-19 vaccine booster dose for all persons aged ≥18 years, 6 months after receipt of a primary mRNA vaccination series or 2 months after receipt of a primary Janssen vaccine dose. During January, 2022, CDC updated booster recommendations, shortening the interval from 6 months to 5 months for receiving an mRNA booster dose after a primary mRNA vaccination series. In January, 2022, CDC also expanded eligibility of booster doses, recommending that adolescents aged 12–17 years receive a booster dose 5 months after a primary Pfizer-BioNTech vaccination series. https://www.cdc.gov/vaccines/covid-19/clinical-considerations/covid-19-vaccines-us.html (Accessed March 27, 2022).

† https://www.cdc.gov/coronavirus/2019-ncov/vaccines/recommendations/immuno.html (Accessed March 27, 2022).

§ CDC’s NHSN provides health care facilities, such as skilled nursing and long-term care facilities with a platform for reporting outcomes and process measures. COVID-19 related vaccination and surveillance data are reported to NHSN through the long-term care facilities COVID-19 Module. https://www.cdc.gov/nhsn/ltc/covid19/index.html

¶ https://covid.cdc.gov/covid-data-tracker/#variant-proportions (Accessed April 4, 2022).

**
https://www.cms.gov/files/document/qso-20-38-nh-revised.pdf

†† NHSN COVID-19 surveillance and vaccination coverage data could not distinguish between immunocompromised nursing home residents who received an additional primary dose and residents who received a booster dose. As such, the category of residents who were vaccinated with an additional or booster dose included residents who received 1) 2 primary mRNA doses followed by a booster dose, 2) 3 primary mRNA doses, 3) 3 primary mRNA doses followed by a booster dose (i.e., 4 total doses), 4) 1 primary Janssen dose followed by a booster dose, 5) 1 primary Janssen dose and an additional primary mRNA vaccine dose, or 6) 1 primary Janssen dose and an additional primary mRNA vaccine dose followed by a booster dose. https://www.cdc.gov/vaccines/covid-19/clinical-considerations/interim-considerations-us.html (Accessed March 27, 2022).

§§ Of 89,969 weekly reports providing resident data, 438 (0.5%) reports from 73 SNFs were excluded because they reported no additional or booster dose coverage throughout the study period.

¶¶
https://www.cms.gov/files/document/qso-20-39-nh-revised.pdf

***
https://www.atsdr.cdc.gov/placeandhealth/svi/index.html

††† 45 C.F.R. part 46, 21 C.F.R. part 56; 42 U.S.C. Sect. 241(d); 5 U.S.C. Sect.552a; 44 U.S.C. Sect. 3501 et seq.

§§§ https://www.cdc.gov/coronavirus/2019-ncov/vaccines/stay-up-to-date.html (Accessed April 20, 2022).

¶¶¶
https://www.medrxiv.org/content/10.1101/2022.01.25.22269843v1

**** https://www.cdc.gov/nhsn/covid19/ltc-vaccination-dashboard.html (Accessed April 11, 2022).

††††
https://www.medrxiv.org/content/10.1101/2022.03.11.22272140v1

§§§§ On March 29, 2022 the Food and Drug Administration authorized a second booster dose of either Pfizer-BioNTech or the Moderna mRNA COVID-19 vaccines, ≥4 months after receipt of a first booster dose for adults aged ≥50 years and persons who are moderately or severely immunocompromised. https://www.fda.gov/news-events/press-announcements/coronavirus-covid-19-update-fda-authorizes-second-booster-dose-two-covid-19-vaccines-older-and
